# Distinct Mutation Patterns Reveal Melanoma Subtypes and Influence Immunotherapy Response in Advanced Melanoma Patients

**DOI:** 10.3390/cancers12092359

**Published:** 2020-08-20

**Authors:** Franz J. Hilke, Tobias Sinnberg, Axel Gschwind, Heike Niessner, German Demidov, Teresa Amaral, Stephan Ossowski, Irina Bonzheim, Martin Röcken, Olaf Riess, Claus Garbe, Christopher Schroeder, Andrea Forschner

**Affiliations:** 1 Institute of Medical Genetics and Applied Genomics, University Hospital Tübingen, 72076 Tübingen, Germany; franz.hilke@charite.de (F.J.H.); axel.gschwind@uni-tuebingen.de (A.G.); german.demidoc@uni-tuebingen.de (G.D.); stephan.ossowski@med.uni-tuebingen.de (S.O.); olaf.riess@med.uni-tuebingen.de (O.R.); 2 Derpartment of Dermatology, Venerology and Allergology, Charité–Universitätsmedizin Berlin, 10117 Berlin, Germany; 3 Image-Guided and Functionally Instructed Tumor Therapies (iFIT) Cluster of Excellence (EXC 2180), University of Tübingen, 72076 Tübingen, Germany; tobias.sinnberg@med.uni-tuebingen.de; 4 Department of Dermatology, University Hospital Tübingen, 72076 Tübingen, Germany; heike.niessner@med.uni-tuebingen.de (H.N.); teresa.amaral@med.uni-tuebingen.de (T.A.); martin.roecken@med.uni-tuebingen.de (M.R.); claus.garbe@med.uni-tuebingen.de (C.G.); andrea.forschner@med.uni-tuebingen.de (A.F.); 5 Portuguese Air Force Health Care Direction, 1649-020 Lisbon, Portugal; 6 German DFG NGS Competence Center, NCCT, 72076 Tübingen, Germany; 7 Institute of Pathology and Neuropathology, University Hospital Tübingen, 72076 Tübingen, Germany; irina.bonzheim@med.uni-tuebingen.de

**Keywords:** Genome of advanced melanoma, acral, mucosal, uveal, melanoma of unknown origin, tumor mutation burden, TMB, immune checkpoint inhibitors, next-generation sequencing

## Abstract

The detection of somatic driver mutations by next-generation sequencing (NGS) is becoming increasingly important in the care of advanced melanoma patients. In our study, we evaluated the NGS results of 82 melanoma patients from clinical routine in 2017. Besides determining the tumor mutational burden (TMB) and annotation of all genetic driver alterations, we investigated their potential as a predictor for resistance to immune checkpoint inhibitors (ICI) and as a distinguishing feature between melanoma subtypes. Melanomas of unknown primary had a similar mutation pattern and TMB to cutaneous melanoma, which hints at its cutaneous origin. Besides the typical hotspot mutation in *BRAF* and *NRAS*, we frequently observed *CDKN2A* deletions. Acral and mucosal melanomas were dominated by CNV alterations affecting *PDGFRA*, *KIT*, *CDK4*, *RICTOR*, *CCND2* and *CHEK2*. Uveal melanoma often had somatic SNVs in *GNA11/Q* and amplification of *MYC* in all cases. A significantly higher incidence of *BRAF* V600 mutations and *EGFR* amplifications, *PTEN* and *TP53* deletions was found in patients with disease progression while on ICI. Thus, NGS might help to characterize melanoma subtypes more precisely and to identify possible resistance mechanisms to ICI therapy. Nevertheless, NGS based studies, including larger cohorts, are needed to support potential genetic ICI resistance mechanisms.

## 1. Introduction

In the last decade, multiple large-scale sequencing studies have elucidated the genetic landscape of cutaneous melanoma and led to the classification of the genetic subtypes *BRAF* mutant, *RAS* mutant, *NF1* mutant or triple wild-type melanoma [1]. In conjunction with earlier exome sequencing studies [2,3], it was shown that the RTK, RAS/RAF/ERK, PI3K and cell cycle pathways were the significant drivers for the oncogenesis of cutaneous melanoma. Additionally, these comprehensive sequencing studies have revealed the genetic landscape not only for cutaneous melanoma but also for acral and mucosal melanoma [4], resulting in the identification of further driver alterations and highlighting the impact of copy number changes in these subtypes [5,6].

Beyond the genetic characterization, the identification of predictive and prognostic markers for treatment responses became a primary focus over the past years [7,8]. Especially in the light of treatment failure in patients treated with immunotherapy, certain genes and signaling cascades, like an amplification of *MDM2* and *EGFR* [9,10,11], *PTEN* deletions and loss of functions mutations in *JAK2* or the interferon gamma signaling were identified as potential markers of primary or acquired resistance [12,13,14,15]. Also, the recent work on the resistance mechanisms in melanoma patients treated with *BRAF* or *MEK* inhibitor therapy revealed new therapeutic strategies [7,8,16].

Therefore, we analyzed the results of next-generation sequencing of 82 patients with advanced melanoma, whose primary tumors or metastases had been sequenced as part of routine clinical care in 2017. We display the diversity of genetic mutations and affected pathways among this clinical cohort, highlighting both genetic differences and similarities among the melanoma subtypes and show potential genetic resistance mechanisms to ICI. We aimed at identifying mutational patterns revealing the origin of melanomas of unknown primary or predicting response to ICI.

## 2. Materials and Methods

### 2.1. Patients and Tumor Tissue

We included all melanoma patients of our Center for Dermatooncology, whose tumors had been sequenced in 2017 as part of routine clinical care. All patients had been informed in a personal conversation with their dermato-oncologist and human geneticist about the procedure of tumor sequencing. Written consent was obtained from all patients. Clinical data were obtained from the patients’ records. All patients had given their consent for their data to be made available for research. The local ethics committee of the University Tuebingen approved this study (approval number 103/2018BO2). The formalin-fixed paraffin-embedded tissue used for sequencing consisted of the latest available tissue, usually metastases that had been removed recently. Genomic DNA was extracted from macrodissected 5µm paraffin sections using the Maxwell^®^ RSC DNA FFPE Kit and the Maxwell^®^ RSC Instrument (Promega, Madison, WI, USA) according to the manufacturer’s instructions.

### 2.2. Sequencing

The sequencing panel was applied in three different versions, targeting 336 cancer-associated genes in the primary setting (ssSCv2; Table S1), 677 (ssSCv3; Table S2) or 693 (ssSCv4; Table S3) genes as well as intronic regions for the detection of distinct fusions in the updated versions (Table S4). As the sequencing panel changed in the course of this study, we focused the analysis on the 275 common genes present in all versions. All samples were sequenced on an Illumina device (e.g., NextSeq500, Illumina Inc., San Diego, CA, USA). Data processing was done by an in-house analysis pipeline (megSAP, https://github.com/imgag/megSAP) using the open source tool BWA for mapping [17] and strelka2 for somatic variant detection [18]. The detection of somatic copy number alterations was done by our in-house software tool clinCNV [19]. Variants were annotated using SnpEff / SnpSift [20,21] and the publicly available Cancer Genome Interpreter [22] for information about driver alterations. The R package Maftools version 2.0.16 was used for visualization [23].

### 2.3. Tumor Mutational Burden

Tumor mutation burden (TMB) was calculated as the number of all somatic alterations (coding SNVs and INDELs) based on the target size of the used panel. Since all three panel versions are designed to detect driver mutations in known tumor suppressor and oncogenes, we had to adjust the calculation to avoid overrating the TMB. The Formula is:

$$\mathrm{TMB}=\left[\frac{\left(\frac{Somatic\;-\;Known\_Tumorgenes}{Target\;size}\;\times Genome\;size\right)+Tumorgenes}{Genome\;size}\right]$$

### 2.4. Gene Set Enrichment Analysis

The gene set enrichment was performed using the clinical feature *histopathological subtype* associated with the samples. The enrichment analysis was done using the gene set enrichment function of the R package MAFTOOLs Version 2.0.16 [23]. It performs various groupwise and pairwise comparisons to identify enriched mutations for every category within a clinical feature.

### 2.5. Statistics

Statistical analysis was performed using the statistical program for social sciences SPSS version 25 (IBM, Edicott, NY, USA) as well as Graphpad Prism Version 8.3.0 (GraphPad Software, LLC, San Diego, CA, USA). The distribution of TMB, SNVs and CNVs was compared between the different melanoma types using Mann–Whitney U test. The overall survival was calculated using the overall survival function in GraphPad Prism Version 8. Follow-up time was defined as the time between the initiation of immune checkpoint inhibition (ICI) and the date of the last follow-up or death from any cause.

## 3. Results

**Figure 1:**
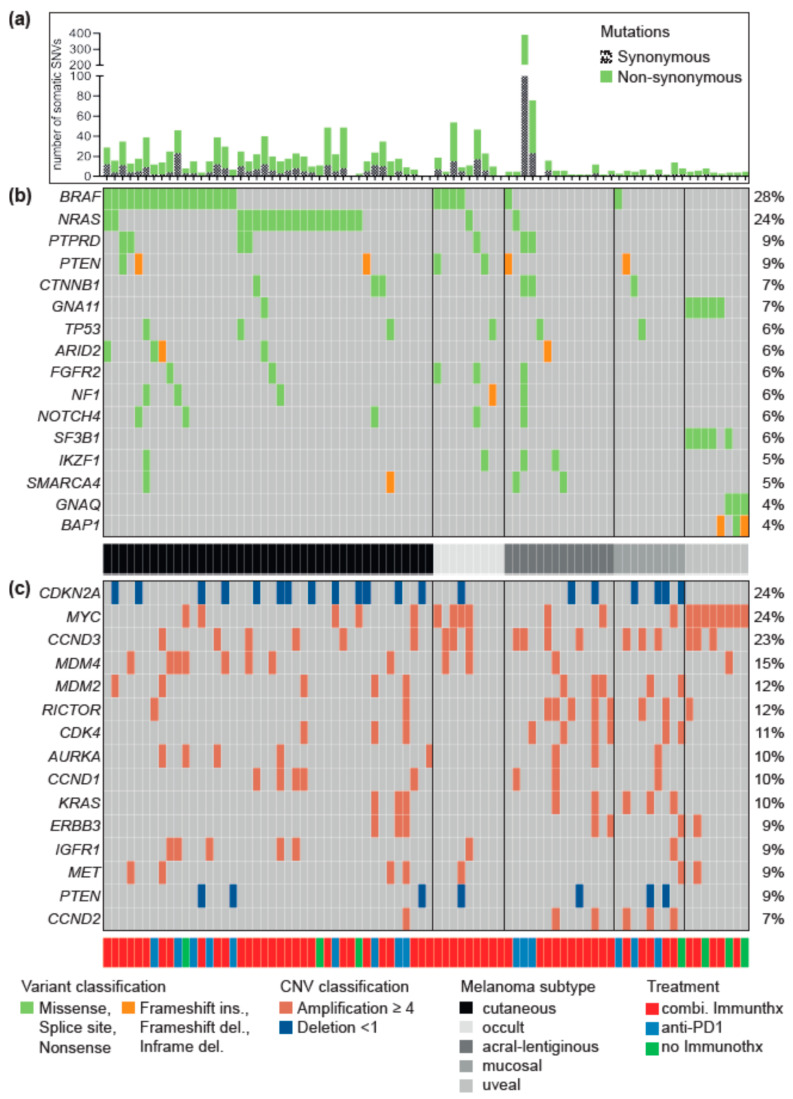
Oncoplot of the most frequently mutated genes with driver mutations. Figure 1 shows a summary of the most frequent driver mutations of the melanoma cohort based on the 275 genes contained in all 3 panel versions. The Oncoplot shows the patients in a horizontal orientation and the gene and the corresponding driver mutations per patient in the vertical orientation. The plot is divided into three parts, in the upper area (**a**) all somatic single nucleotide variants (SNVs) and small insertions and deletions (INDELs) per patient, including the synonymous variants, are shown. The middle panel (**b**) summarizes all somatic SNVs found with a frequency of at least five percent, plus the two genes *GNAQ* and *BAP1*. The lower panel (**c**) summarizes the 15 most mutated genes with somatic copy number changes (CNVs). For both panel (**b**) and (**c**), only mutations that were annotated as drivers or druggable biomarkers by the Cancer Genome Interpreter (CGI) are shown. Colors indicate different mutation types (s. legend for details). The entire cohort is sorted by the histopathological subtype in a grayscale (cutaneous, acral-lentiginous, mucosal, uveal, melanoma of unknown primary = occult). In addition, the type of therapy is annotated (red: combined immunotherapy, n = 60; blue: anti-PD-1, n = 15; green: no immunotherapy, n = 7).

In total, 82 tumor tissue samples from 82 different patients were sequenced and analyzed. The cohort included patients with cutaneous melanoma (n = 42), acral (n = 14), mucosal (n = 9) and uveal melanoma (n = 8), as well as melanoma of unknown primary (n = 9). The gender distribution was almost equal, with 37 (45%) females and 45 (55%) males. The median age at the time of primary melanoma diagnosis was 57 years (IQR 46–67 years). The majority of patients (n = 75) had been treated with ICI, either with the PD-1 antibodies nivolumab and pembrolizumab alone and/or nivolumab in combination with ipilimumab, a CTLA-4 antibody (Figure 1, Table 1).

### 3.1. Driver Alterations and Signaling Cascade

Comprehensive sequencing of the 82 tumor samples identified in total 1650 somatic variants (SNVs and INDELs) and 2.137 somatic copy number alterations, such as amplification or deletion of a complete gene (Tables S5 and S6). We obtained a median number of 10 SNVs/INDELs per patient and a median TMB of 5.82 (range: 0–151.75; Table S7). Furthermore, we referred to CancerGenomeInterpreter.org for the annotation of driver mutations and revealed a total number of 527 driver alterations, 283 SNVs and 244 CNVs, affecting 149 genes (supplement Tables S8 and S9).

The genomic classification of our cohort into *BRAF*-, *RAS*-, *NF1*-mutated or triple-wildtype showed that more than half of the patients carried a driver mutation in either one of the three genes (Figure 1b). The serine/threonine kinase *BRAF* was the most frequently mutated gene of this cohort (28%). The vast majority were hotspot mutations V600E/K/R (91%). Also, one patient had the *BRAF* G466E driver mutation, and another had two simultaneous driver mutations at positions P367S and K601E. The second most frequent mutation was the GTPase *NRAS* (24%) with Q61K/L/R mutations. In two patients, we observed the rare combination of simultaneously occurring hotspot mutations in the genes *BRAF* (V600E) and *NRAS* (Q61K and R, respectively). Moreover, hotspot mutations in *HRAS* (Q61R) and *KRAS* (G12D, D119H) were present in one, two patients, respectively (not shown in Figure 1). Driver mutations in the gene *NF1* were rare with 6%, while three of the patients either had a *BRAF* or *NRAS* hotspot mutation. The rest of the cohort, 36 patients (44%), were classified as triple wild-type. Besides driver mutations in the genes *BRAF*, *NRAS* and *NF1*, we found frequent activating amplifications in the receptor tyrosine kinases *ERBB3* and *MET*, as well as in the signal transducers *KRAS* and *PTPRD* (Figure 1c). Thus, the RTK/RAS/MAP kinase signaling pathway (hereafter RTK/RAS) is most frequently affected by driver mutations in our cohort (79%). Interestingly, the activating mutations in *ERBB3*, *MET* and *KRAS* were more abundant in the triple wild-type group.

The genes most frequently affected by copy number alterations (deletion or amplification) were *CDKN2A* (24%) and *CCND3* (23%), both of which belong to the cell cycle control (Figure 1c). Activating *MYC* amplifications were also detected in 24% of the patients. The PI3K signaling cascade was the third most frequent mutated signaling pathway (38%) with driver mutations in the genes *PTEN* (17%) and *RICTOR* (12%). Furthermore, we found frequent driver mutations in the Wnt and SWI/SNF signaling pathway (*CTNNB1*, *ARID2*, and *SMARCA4*).

### 3.2. Differences between Melanoma Subtypes

**Figure 2:**
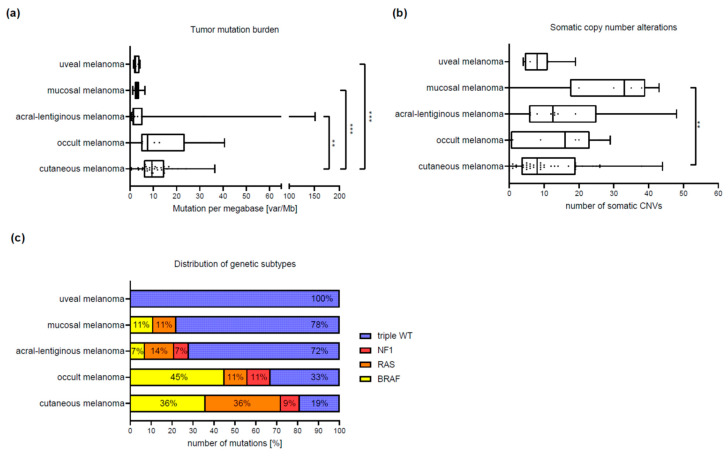
Tumor mutation burden, copy number variants and genetic subtypes. Figure 2 shows the comparison of tumor mutation burden (TMB), number of somatic copy number changes (CNVs) and the frequency of the genetic subtypes *BRAF*, *NRAS*, *NF1* and Triple wild-type (triple-WT) between the four histopathological subtypes (cutaneous, acral-lentiginous, mucosal, uveal) and the melanoma of unknown primary (occult). The figure consists of 3 histograms (**a**–**c**), whereby the y-axis always represents the histopathological subtypes. On the x-axis the TMB is indicated in (**a**), as mutations per megabase (Mut/Mb), in (**b**) the number of somatic CNVs, in absolute numbers related to the 275 genes analyzed and in (**c**) the genetic subtype in absolute numbers. Patients with cutaneous melanoma have the most somatic SNVs and thus the highest median TMB compared to the remaining three subtypes (*p* ≤ 0.003). The median TMB of occult and cutaneous melanoma is only slightly different with 7.5 var/Mb versus 9.4 var/Mb, respectively. The mucosal melanoma clearly has the most somatic CNVs with a median of 33 and there is a significant difference between the mucosal and cutaneous melanoma (*p* = 0.025) and the uveal and occult melanoma (*p* = 0.025). Cutaneous melanoma shows an even distribution of *BRAF* and *RAS* mutated patients (40% each) and patients with melanoma of unknown primary are also in 45% *BRAF* mutated. The other three subtypes show a clear overweight of tumors with the status triple-WT.

As part of the further analysis, we attempted to identify characteristic genetic differences between the five histopathological melanoma subtypes, based on panel sequencing. Interestingly, we found great similarities between the cutaneous melanoma and melanoma of unknown primary. Both subtypes were dominated by somatic SNVs, leading to the highest number of mutations (median = 7) and a significantly higher TMB (median = 9.4, range = 0–36.4; *p* < 0.003) in patients with cutaneous melanoma compared to the other subtypes (Figure 2a, Table 2). The majority of the cutaneous and melanoma of unknown primary could be classified in the genomic subtypes *BRAF*, *RAS* and *NF1*. Only 19% of cutaneous or 33% melanoma of unknown primary were triple wild-type. In contrast, patients with acral and mucosal melanoma were triple wild-type in most of the cases (acral = 72%, mucosal = 78%) and had CNVs predominantly. In patients with mucosal melanoma, we observed significantly more somatic CNVs (median = 33, range 0–43; *p* < 0.002) than in cutaneous melanoma (Figure 2b, Table 2).

**Figure 3:**
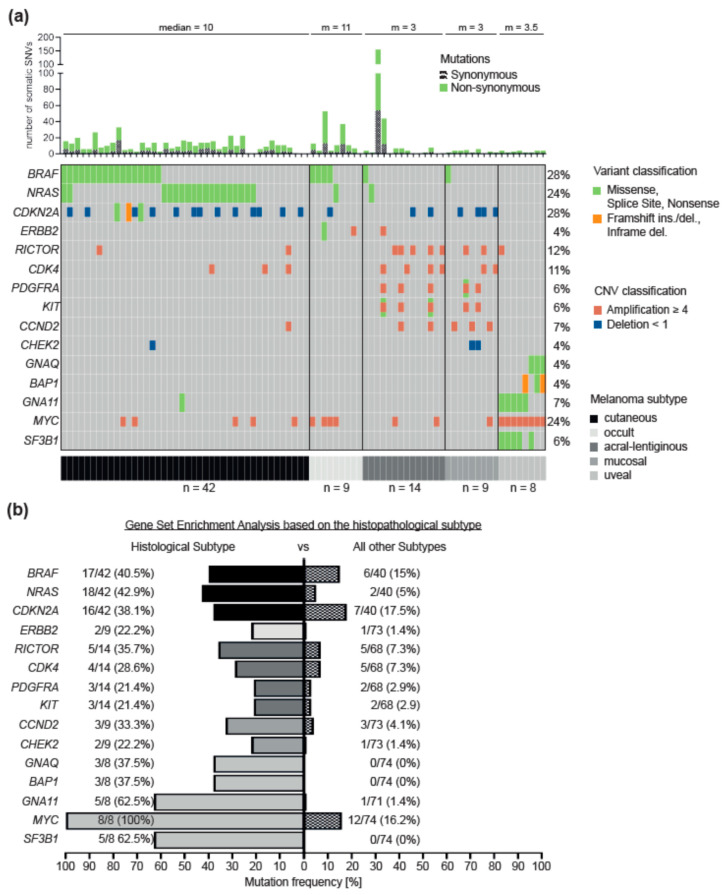
Gene set enrichment analysis of histopathological subtypes. Figure 3 summarizes the genetic differences, based on the gene set enrichment analysis (subtype versus rest of the cohort), of the four histopathological subtypes and the melanoma of unknown primary (occult). The figure consists of a histogram (**a**) showing the number of somatic changes (SNVs), including synonymous variants and the median (m) of each subtype. Below is an Oncoplot. It is sorted by histopathological subtypes cutaneous, acral-lentiginous, mucosal, occult and uveal melanoma (gray scale). It summarizes the genes that are significant (*p* < 0.05) for each subtype. In vertical alignment, the patients are summarized and in horizontal alignment the genes and the corresponding changes. On the left side the frequency, as a histogram in percent, of the somatic mutations (drivers) per gene, related to the total cohort, is shown. The panel (**b**) shows the frequency (in percent) of mutations per gene related to the subtypes (gray scale) compared to the rest of the cohort. For cutaneous melanoma, the genes *BRAF* (*p* = 0.009), *NRAS* (*p* = 0.0004) and *CDKN2A* (*p* = 0.03) are significantly enriched mutated compared to the rest of the cohort. For the acral-lentiginous melanoma the genes *RICTOR* (*p* = 0.01), *CDK4* (*p* = 0.04), *PDGFRA* (*p* = 0.03) and *KIT* (*p* = 0.03) are enriched mutated, for the mucosal melanoma the genes *CCND2* (*p* = 0.01) and *CHEK2* (*p* = 0.03) are enriched mutated, for the melanoma of unknown primary the gene *ERBB2* (*p* = 0.03) is enriched mutated and for the uveal melanoma the genes *GNAQ* (*p* = 0.0006), *GNA11* (*p* = 0.00001), *BAP1* (*p* = 0.0006), *MYC* (*p* = 0.000003) and *SF3B1* (*p* = 0.0000002) are enriched mutated.

To identify characteristic genetic differences between the histopathological melanoma subtypes, we performed a gene set enrichment analysis (Figure 3, Tables S10 and S11), revealing several significant differences. In contrast, uveal melanoma is the most distinct subtype. Compared to cutaneous, acral and mucosal melanoma, there was no enrichment of mutations in the RTK/RAS signaling pathway, but frequent mutually exclusive mutations in the genes of the guanine nucleotide-binding protein subunits *GNA11* and *GNAQ* in combination with either the splicing factor *SF3B1* or the deubiquitinating enzyme *BAP1* (Figure 3a,b). Of note, we found *MYC* amplifications in each of the eight uveal melanoma patients, which is a significant difference to the other melanoma subtypes (*p* < 0.05).

On the other hand, hotspot mutations in the genes *BRAF* and *NRAS*, in combination with homozygous deletions of the gene *CDKN2A*, were frequently found in cutaneous melanoma. In contrast, acral and mucosal melanoma were dominated by copy number changes affecting the RTK/RAS pathway and regulators of the cell cycle (Figure 3a). The co-amplifications of the receptor tyrosine kinases *KIT* and *PDGFRA* on chromosome 4, as well as *CDK4* on chromosome 12, are of particular interest for acral melanoma. Mucosal melanoma had a significant accumulation of amplification in the regulator of the cell cycle control *CCND2* (Figure 3b). Furthermore, a member of the Fanconi anemia pathway, *CHEK2*, was enriched for deletions in mucosal melanoma.

### 3.3. Characteristics of Patients Treated with Immune Checkpoint Inhibitors

**Figure 4:**
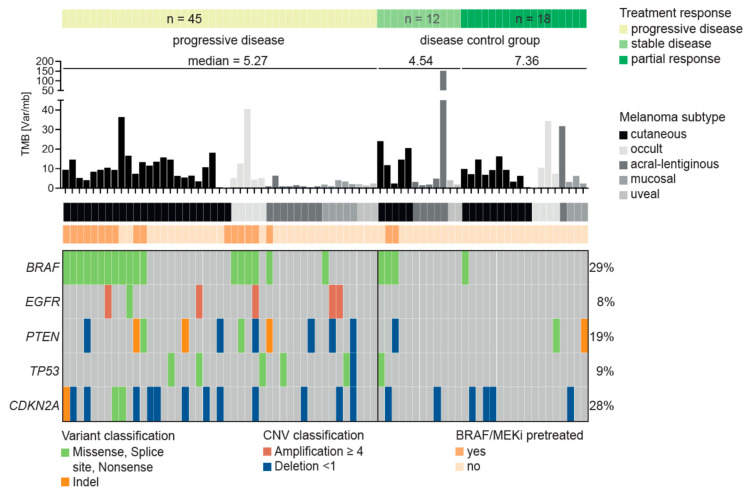
Predictor for resistance to immune checkpoint therapy. Figure 4 shows the result of the Fisher’s test identifying a genetic predictor for the resistance to immune checkpoint inhibitor therapy based on the RECIST response classification (progressive disease versus disease control [stable disease, partial response]). Mutations in three genes—*EGFR*, *PTEN* and *TP53* - were identified as individual predictors for a progressive disease at the first staging. In 89% of cases (16 out of 18), patients with *BRAF* mutations had previously received BRAF/MEK inhibition before receiving immunotherapy. Together the mutations and the failed BRAF/MEKi pre-therapy predict 82% of the patients as PD (*p* = 0.007).

The majority of our cohort had been treated with ICI (75 of 82). Most of the patients (n = 60) had received combined immunotherapy with nivolumab and ipilimumab, and 15 patients had been treated only with PD-1 antibodies alone. In order to identify possible genes associated with resistance to ICI therapy, we classified treatment response according to RECIST 1.1 [24] in three groups: progressive disease (PD) (n = 45), stable disease (SD) (n = 12) and partial response (PR) (n = 18). Interestingly, most of the patients with PD and a *BRAF* mutation (16 of 18) had received therapy with BRAF and MEK inhibition (BRAF/MEKi) before initiation of ICI compared to those with stable disease or partial response (2 of 4) (Figure 4). As expected, the poorest survival was achieved by patients with PD, followed by patients with SD, while the best survival was that of patients with PR (Figure S1a). The one-year overall survival (OS) rate was 51% for patients with PD compared to 83% for SD and 100% for PR (Table S12).

The comparison of the OS between the three groups of patients, i.e., (a) melanoma *BRAF* wild-type, (b) *BRAF* mutant (*BRAF*^mut^) treatment naïve and (c) pretreated with *BRAF/MEK*i (before ICI) melanoma showed a significantly better OS for patients in the first two groups (*p* = 0.01) (Figure S1b, Table S13). Similarly, the one-year OS rate was 80% for *BRAF*-WT and 72% for treatment naïve *BRAF*^mut^ patients compared to only 63% in patients who received prior therapy with a *BRAF/MEK*i.

### 3.4. Resistance Predictor for Immune Checkpoint Inhibitor Therapy

We next aimed at developing an ICI resistance predictor based on known resistance mechanisms. For instance, mutations in *PTEN* and amplifications of *EGFR* have previously been linked to resistance to ICI [25]. Mutations in *TP53* have been associated with prolonged progression-free survival in lung cancer after immunotherapy [25], but also to increased ICI resistance in melanoma [26]. Indeed, we observed in our cohort that mutations in *PTEN* and *EGFR* are enriched in the PD group (24.5% and 13.3%) compared to the SD+PR group (10% and 0%), although only the difference for *EGFR* is borderline significant. As previously suggested for melanoma, we also found that *TP53* mutations are enriched in the PD (13.3%) compared to the SD+PR group (3.3%). As described above, we further observed that pretreatment with BRAF/MEKi is associated with PD (*p* = 0.01).

Combining these features in a multi-gene predictor of resistance to ICI, we observed that patients who are either pretreated with BRAF/MEKi or have a mutation in at least one of the genes *PTEN*, *EGFR* or *TP53* are highly likely to show PD (84.4%, *p* = 0.0002; Table S14). Accordingly, the relative risk (RR) to show PD in the first staging after starting with ICI was 1.99 for cases in which the tumor harbors a mutation in *EGFR, PTEN* or *TP53* or the patient was pretreated with BRAF/MEKi. However, the sensitivity of the predictor is rather low, identifying 62% of PD patients (28/45), but with a good specificity of 80% (24/30). We also tested if a mutation in at least one of the genes *PTEN*, *EGFR* or *TP53* alone (excluding BRAF/MEKi pretreatment as a feature) is a predictor of PD, which indeed is the case (82% show PD, *p* = 0.007), although only 42% of the PD patients (19/45) are identified with this reduced combination (RR = 1.65).

We have previously reported that deletions or loss of function mutations in *CDKN2A* increase the resistance to ICI [26]. Similarly, we find that *CDKN2A* deletions or loss of heterozygosity are enriched in PD (*p* = 0.024). However, mutations are mostly redundant to the already included genes, i.e., many patients with *CDKN2A* deletion also harbor a mutation in *PTEN*, *EGFR* or *TP53*. Still, the inclusion of *CDKN2A* increases the sensitivity of predicting PD to 73.3% (33/45 and RR = 2.05) but reduces specificity to 66.7% (20/30). Another well-known resistance gene, *JAK2* [13,27], was only mutated in three patients, two of which additionally harbored a *PTEN* or an *EGFR* mutation. The highest specificity of 100% (30/30) is observed for any combination of the above changes in *EGFR, PTEN, TP53* and *CDKN2A*. All nine patients with such a combination showed PD in the first staging, although the latter has reduced sensitivity (20% or 9/45). The absence of combined changes in these four genes could be associated with response to ICI, while the occurrence of such a combination seems to be highly predictive for a poor response (RR = 1.83). The high number of unidentified progressions of ICI by this combined predictor indicates additional factors that influence ICI response.

## 4. Discussion

In this study, we aimed to evaluate TMB and driver mutations in a real-world cohort of patients with cutaneous, acral, mucosal and uveal melanoma and melanoma of unknown primary. Employing a targeted cancer panel approach, we found distinct genetic patterns for different histopathological subtypes. All subtypes were characterized by driver mutations in the genes of the RTK/RAS signaling pathway as well as cell cycle control and the PI3K pathway, except for patients with uveal melanoma. This subtype was dominated by amplification of *MYC* and *GNA11* or *GNAQ* mutations in combination with the genes *BAP1* or *SF3B1*. The high frequency of *MYC* amplification has been described in previous works, and it is known that high expression of *MYC* in tumors is associated with an increased risk of developing metastases and a worse prognosis [28,29,30,31,32]. Furthermore, we found mutual exclusivity of *SF3B1* or *BAP1* driver mutations in all patients with uveal melanoma. The splicing factor *SF3B1* is associated with an increased risk of metastasis and the deubiquitinase *BAP1* with a worse prognosis [32,33]. These observations are in line with the clinical status of the included patients. All of them were already metastasized and characterized as advanced uveal melanomas.

The other three subtypes, as well as melanoma of unknown primary, were, as mentioned above, dominated by driver mutations in the genes of the cell cycle control (*CDKN2A*) and the RTK/RAS signaling pathway (*BRAF*, *NRAS*, *ERRB2*). Besides, cutaneous and melanoma of unknown primary had the highest tumor mutation burden compared to the other subtypes. Interestingly, they are also similar in terms of the number of CNVs and the frequency of genomic subgroups, *BRAF-*, *RAS-*, *NF1*-mutated or triple wild type. Taken together, these results support the assumption that the cutaneous subtype and melanoma of unknown primary are similar and that the latter probably originates from regressed or unrecognized primary cutaneous melanomas [34,35].

In comparison, the number of somatic CNVs was higher in acral and mucosal melanoma than in cutaneous melanoma. This result corresponds to the current state of knowledge [36,37], whereby in our cohort, a significantly higher number of CNVs could only be detected in mucosal melanoma. The driver mutations in the genes *RICTOR*, *CDK4*, *PDGFRA*, *KIT* were specific for acral melanoma, while the amplification or deletion of the genes *CCND2* and *CHEK2* are uniquely found in mucosal melanoma. Our study confirms the observations reported in other recent studies [4,5,6].

Furthermore, the comparison of driver gene mutations in patients with PD compared to patients with SD+PR under ICI treatment revealed enrichment of *EGFR*, *PTEN* and *TP53* gene mutations together with *BRAF* V600 mutations, if pretreated with BRAF/MEKi. We observed an 84.4% chance of progressive disease under ICI (*p* = 0.0002) for cases where the tumor harbors a mutation in one of these genes, or the patient was pretreated with BRAF/MEKi. Hence, the good performance of our multi-gene predictor supports previous observations and shows that the combined analysis of resistance mechanisms might be a powerful tool for a treatment recommendation. However, it is necessary to apply the predictor to an independent cohort in future studies to robustly evaluate its precision and recall. Moreover, other features such as TMB or additional genes could be incorporated to improve the predictor’s sensitivity, although again, independent testing would be required. By combining similar studies, one might soon reach the critical mass to train machine learning based classifiers such as random forests, facilitating the inclusion of more features. Moreover, a large homogeneous cohort of a single melanoma subtype treated with ICI would very likely improve the accuracy of a trained predictor. Several studies in non-small-cell lung cancer have already shown the ineffectiveness of ICI in the case of tumors with *EGFR* mutations and low TMB [38,39,40]. Also, several retrospective studies have shown that the outcome of ICI is improved if these patients do not receive prior BRAF/MEKi therapy [41,42,43]. Moreover, an increased risk of progressive disease at the first staging was associated with the presence of *PTEN* and *TP53* driver mutations. Both, if mutated, are potential genetic markers for resistance to ICI [26,44].

Besides, driver mutations in the cyclin-dependent kinases *CDKN2A*, *CDK4*, *CCND1/2/3* and the genes *MDM2/4* and *TP53* were abundant, which presumably contribute to significant deregulation of cell cycle control. Deletions of gene *CDKN2A* appear to lead to resistance to immunotherapy and, in combination with the deregulation of *CDK4*, worsen prognosis in acral melanoma [13,45]. Therefore, the introduction of *CDK4/6* inhibitors could broaden treatment options [45,46,47]. Combined treatment with *MEK* and *CDK4/6* inhibitors could also be important [48]. Another potential treatment targets are driver mutations in the genes *ATM*, *CHEK2*, *FANCA*, *FANCC* and *BRCA2*, which belong to the DNA repair signaling pathway. One treatment option would be *PARP* inhibitors or platinum-based chemotherapy [49].

One of the limitations of the study is the fact that the evaluated cohort includes patients treated in daily clinical practice without further specific inclusion criteria. This means that all patients included were in an advanced stage of the disease, including distant metastases, and most of them had already received several systemic therapies. With regards to the differences between the melanoma subtypes [50,51] and the identification of potential druggable targets, this is probably not that relevant. While there might be differences between primary melanoma and metastasis, it still seems to be appropriate to tailor the therapy regime to the genomic pattern of the metastases. Concerning the advanced tumor stages of the patients and prior systemic therapies, the interpretation of genetic patterns for ICI resistance might be difficult. Sequencing the tumor tissue at an earlier stage without pretreatment and at the time of progression might be more appropriate to detect possible genetic resistance mechanisms.

Finally, we observed that the TMB of cutaneous melanoma in our cohort was lower than that of TCGA [1]. This difference may be because the estimated TMB of our cohort is based on a panel of genes and, therefore, different from the whole-exome approach used in the TCGA cohort. Furthermore, we probably have a negative selection of patients in our cohort as most of our patients had not responded to ICI, which is likely to be more common when the TMB is lower [50,51,52,53].

## 5. Conclusions

NGS might help to characterize melanoma subtypes more precisely and to better understand ICI failure in some points. We observed that mutational signatures (TMB, CNV burden, driver genes) of melanoma of unknown primary resemble cutaneous melanomas. Furthermore, in patients with a progressive disease, we found a strong enrichment of cases with a mutation in at least one of the genes *BRAF*, *EGFR*, *PTEN*, *TP53* or *CDKN2A*. Nevertheless, prospective studies are urgently needed to learn more about potential ICI resistance mechanisms, and further effort should be made to introduce targeted therapies beyond *BRAF*, *MEK* or *KIT* inhibitors, such as *MYC* or *MDM2* inhibitors for metastasized melanoma.

## Figures and Tables

**Table 1:** Clinical characteristics of the 82 patients.

Patient Characteristics	
**Age at the first diagnosis of melanoma**	**years**
**Median**	**57 (46–67)**
**Range**	**17–85**
**Sex**	**no of patients (%)**
Female	37(45)
Male	45 (55)
**Melanoma type**	**no. of patients (%)**
Cutaneous	42 (51)
Acral	14 (17)
Uveal	8 (10)
Mucosal	9 (11)
Occult	9 (11)
**Tumor thickness of primary melanoma**	**mm**
Median (IQR)	3.3 (1.8–5.1)
Range	0.38–5.1
**The tumor stage at the time of tumor sequencing**	**no. of patients (%)**
Stage II	5 (6)
Stage IV	77 (94)
Immunotherapy	76/82 (93)
Targeted therapy	21 (26)
Chemotherapy	4 (5)
No systemic treatment	5 (6)
**Origin of the tissue sequenced**	**no of patients (%)**
Lymph node	19 (23)
Other metastasis	57 (70)
Primary melanoma	6 (7)
**Pretreatment of tissue sequenced**	**no. of patients (%)**
Tissue therapy naïve	52 (63)
Tissue progressive under ICI	18 (22)
Tissue progressive under targeted therapy	8 (10)
Tissue progressive under chemotherapy	4 (5)

**Table 2:** Genetic characteristics of the 82 patients.

Genetic Characteristics of the 82 Patients	Cutaneous	Acral	Mucosal	Uveal	Occult
	(n = 42)	(n = 14)	(n = 9)	(n = 8)	(n = 9)
**Tumor mutation burden (TMB)**					
Median (IQR)	9.4 (6–14.6)	1.5 (1–5.3)	3.2 (2–3.8)	2.8 (1.6–4)	7.5 (4.8–23.5)
Range	0–36.444	0.51–151.8	1.1–6.a	1.5–4.2	0–40.6
**Comparison to cutaneous subtype ^1^**		*p* = 0.0027 *	*p* = 0.0003 *	*p* = 0.0003 *	*p* = 0.837
**Single nucleotide variants (SNVs)**					
Median (IQR)	10 (6–15)	3 (1–7.3)	3 (2–4.5)	3.5 (2–4)	11 (4–25)
Range	0–33	0–156	1–6	2–4	1–44
**Comparison to cutaneous subtype ^1^**		*p* = 0.0134	*p* = 0.0005 *	*p* = 0.0008 *	*p* = 0.995
**Copy number variants (CNVs)**					
Median (IQR)	8 (3.5–19)	12.5 (5.8–25)	33 (17.5–39)	8 (4.5–11)	16 (0.5–23)
Range	0–44	0–48	0–43	4–19	0–29
**Comparison to cutaneous subtype ^1^**		*p* = 0.321	*p* = 0.002	*p* = 0.995	*p* = 0.587

^1^ Mann–Whitney UTest, * significant.

## Data Availability

The datasets used and/or analysed during the current study are available from the corresponding author on reasonable request.

## Supplementary Materials

The following are available online at https://www.mdpi.com/2072-6694/12/9/2359/s1. Figure S1: Shown is the survival of the 75 patients who received immunocheckpoint therapy depending on response group (a) and the influence of BRAF mutation status and previous treatments (b). Table S1: Cancer panel version ssSCv2. Table S2: Cancer panel version ssSCv3. Table S3: Cancer panel version ssSCv4. Table S4: List of common 275 genes present in all three panel versions. Table S5: List of all somatic mutation (SNVs and INDELs) identified in all 82 patients unfiltered for the common 275 genes. Table S6: List of all somatic copy number variants (Amp & Del) identified in all 82 patients unfiltered for the common 275 genes. Table S7: Tumor mutation burden [Mut/Mb] of all 82 patients. Table S8: List of all somatic mutation (SNVs and INDELs) filtered for the common 275 genes and driver mutations. Table S9: List of all somatic copy number variants (Amp & Del) identified in all 82 patients filtered for the common 275 genes and driver mutations. Table S10: genomic subtype. Table S11: gene set enrichment based on histopathological subtype. Table S12: Overall survival based on disease groups. Table S13: Overall survival based on BRAF mutation status and pretreatment with BRAF/MEK inhibitors. Table S14: Fisher’s test Immunotherapy Resistance Predictor.
