# Supplementary figures and images for: Distinct Mutation Patterns Reveal Melanoma Subtypes and Influence Immunotherapy Response in Advanced Melanoma Patients
Source: Cancers (Basel). 2020 Aug 20;12(9):2359. doi: 10.3390/cancers12092359 (PMC7563780; doi:10.3390/cancers12092359)

**(a)**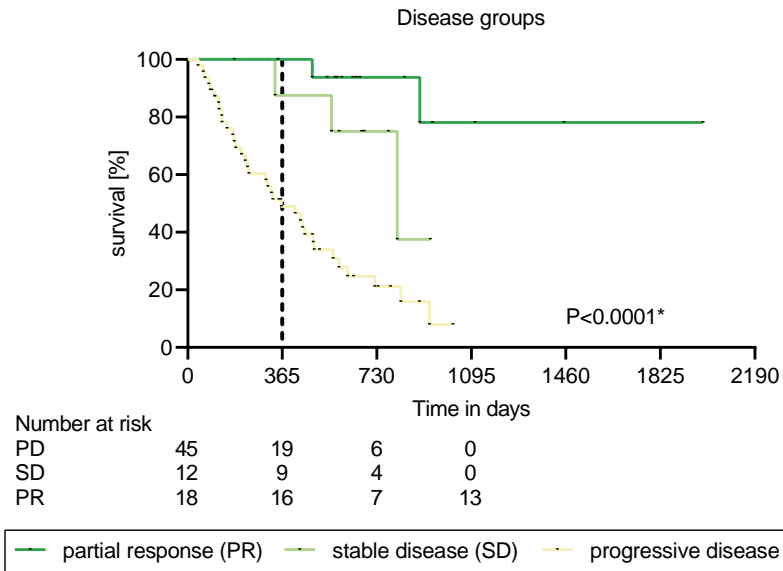**(b)**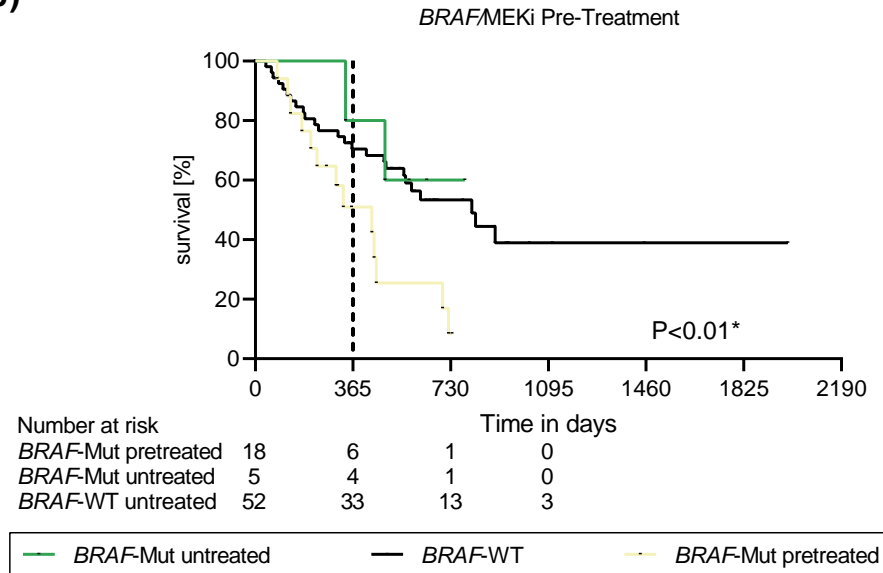

Supplement: Supplementary file 1 [file cancers-12-02359-s001.zip › cancers-898298-supplementary/SupplementaryFigure1_revised.pdf]
